# The Effect of Cell Surface Expression and Linker Sequence on the Recruitment of Arrestin to the GIP Receptor

**DOI:** 10.3389/fphar.2020.01271

**Published:** 2020-08-13

**Authors:** Suleiman Al-Sabah, Lobna Adi, Moritz Bünemann, Cornelius Krasel

**Affiliations:** ^1^Department of Pharmacology and Toxicology, Faculty of Medicine, Kuwait University, Kuwait City, Kuwait; ^2^School of Pharmacy, Institute for Pharmacology and Toxicology, The Philipps University of Marburg, Marburg, Germany

**Keywords:** G protein-coupled receptor, arrestin, fluorescence resonance energy transfer, bioluminescence resonance energy transfer, glucagon-like peptide-1, glucose-dependent insulinotropic polypeptide

## Abstract

The glucose-dependent insulinotropic polypeptide (GIP) and the glucagon-like peptide-1 (GLP-1) receptor are important targets in the treatment of both type 2 diabetes mellitus (T2DM) and obesity. Originally identified for their role in desensitization, internalization and recycling of G protein-coupled receptors (GPCRs), arrestins have since been shown to act as scaffolding proteins that allow GPCRs to signal in a G protein-independent manner. While GLP-1R has been reported to interact with arrestins, this aspect of cell signaling remains controversial for GIPR. Using a (FRET)-based assay we have previously shown that yellow fluorescent protein (YFP)-labeled GIPR does not recruit arrestin. This GIPR-YFP construct contained a 10 amino acid linker between the receptor and a *XbaI* restriction site upstream of the YFP. This linker was not present in the modified GIPR-SYFP2 used in subsequent FRET and bioluminescence resonance energy transfer (BRET) assays. However, its removal results in the introduction of a serine residue adjacent to the end of GIPR’s C-terminal tail which could potentially be a phosphorylation site. The resulting receptor was indeed able to recruit arrestin. To find out whether the serine/arginine (SR) coded by the *XbaI* site was indeed the source of the problem, it was substituted with glycine/glycine (GG) by site-directed mutagenesis. This substitution abolished arrestin recruitment in the BRET assay but only significantly reduced it in the FRET assay. In addition, we show that the presence of a N-terminal FLAG epitope and influenza hemagglutinin signal peptide were also required to detect arrestin recruitment to the GIPR, most likely by increasing receptor cell surface expression. These results demonstrate how arrestin recruitment assay configuration can dramatically alter the result. This becomes relevant when drug discovery programs aim to identify ligands with “biased agonist” properties.

## Introduction

Glucose-dependent insulinotropic polypeptide (GIP) and glucagon-like peptide-1 (GLP-1) are peptide hormones released from the gut postprandially and act primarily to potentiate glucose-induced insulin secretion (Kim and Egan, 2008). Together these peptides mediate the incretin effect (i.e. the larger insulin response elicited by oral administration of glucose compared to intravenous administration, under comparable plasma glucose levels) by binding to their receptors (GIPR and GLP-1R respectively) expressed on pancreatic β cells (Mcintyre et al., 1964; Nauck et al., 1986). An early characteristic of type 2 diabetes mellitus is an impairment of the incretin effect (Holst et al., 2011). However, at pharmacological concentrations GLP-1 is insulinotropic in patients with T2DM whereas GIP does not appear to be (Nauck et al., 1993). As a result, several GLP-1 analogs are currently used clinically to treat T2DM, but to date, this is not the case for GIPR agonists (Andersen et al., 2018). Interestingly, peptide agonists that target both receptors have been investigated and appear to show greater efficacy in terms of glycemic control and weight loss and fewer adverse effects than agonists that target GLP-1R alone (Frias et al., 2018).

GIPR and GLP-1R are closely related members of the secretin family of G protein-coupled receptors (GPCRs) and share a high degree of sequence homology (Mayo et al., 2003; De Graaf et al., 2016). When activated, both receptors couple through Gs, resulting in an increase in intracellular cAMP (Yabe and Seino, 2011; Al-Sabah, 2016). GLP-1R has also been reported to couple to other G proteins, including Gq, which has been shown to mediate receptor internalization (Montrose-Rafizadeh et al., 1999; Thompson and Kanamarlapudi, 2015). Activation of GLP-1R also results in the rapid recruitment of arrestin to the receptor (Jorgensen et al., 2005). However, this remains a controversial subject with regards to GIPR, with some groups showing no interaction between GIPR and arrestin and others who do (Al-Sabah et al., 2014; Ismail et al., 2015; Gabe et al., 2018). Non-visual arrestins were first identified for their role in the desensitization, internalization and recycling of GPCRs (Lefkowitz, 2005; Reiter and Lefkowitz, 2006). Subsequently, arrestins have been shown to also operate as scaffolding proteins allowing GPCRs to signal in a G protein-independent manner, such as *via* ERK1/2 MAPKs (Luttrell et al., 2001; Xiao et al., 2010). This has led to the concept of biased agonism or functional selectivity, where ligands can favor a G protein-dependent pathway or an arrestin-dependent pathway (Koole et al., 2010; Reiter et al., 2017). This could potentially lead to new therapeutics with greater efficacy and fewer adverse effects (Violin et al., 2014). While there is evidence that arrestins are involved in mediating GLP-1’s insulinotropic effects on pancreatic β-cells, G protein biased GLP-1R agonists have been shown to produce greater long-term insulin release and less nausea than more balanced agonists (Sonoda et al., 2008; Jones et al., 2018).

Arrestin recruitment to GPCRs can be monitored by coimmunoprecipitation or visualization of fluorescently labeled arrestin to the plasma membrane by confocal microscopy. These techniques however are not without their drawbacks. For example, coimmunoprecipitation requires the use of a cross linking agent and neither technique is particularly amenable to quantification. More recently several resonance energy transfer techniques such fluorescence resonance energy transfer (FRET) and bioluminescence resonance energy transfer (BRET) have been developed to study various aspects of GPCR functionality (Krasel et al., 2005; Pfleger et al., 2006). Both intermolecular FRET and BRET require the receptor and arrestin to be fused to a suitable donor/acceptor pair which allows the quantification of rate of arrestin association/dissociation to the receptor and the potency of agonists to recruit arrestin to the receptor (Krasel et al., 2004; Ayoub, 2016). Functional affinity or efficacy of an agonist for a given pathway are required to calculate a ligands’ bias (Jaeger et al., 2014).

In this study we investigate how assay configuration, especially modifying receptor cell surface expression and altering the linker-region between the receptor and fluorescent protein, can dramatically affect the result of both FRET and BRET-based arrestin recruitment assays, potentially leading to false-positive results.

## Materials and Methods

### Construction of cDNA

cDNA encoding the following constructs have been previously described; wild-type and C-terminally enhanced yellow fluorescent protein (eYFP)-labeled human GLP-1R and GIPR (GLP-1R-eYFP, GIPR-eYFP) (Al-Sabah et al., 2014), arrestin3-cyan fluorescent protein (Arr3-CFP) (Krasel et al., 2005), G protein-coupled receptor kinase 2 (GRK2) (Krasel et al., 2001). GIPR- and GLP-1R-labeled at the C-terminus with super yellow fluorescent protein 2 (SYFP2) (Kremers et al., 2006) were generated by amplifying the open reading frame of human GLP-1R and GIPR with primers which added a *HindIII* restriction site ahead of the start codon and replaced the stop codon with an *XbaI* site. The start codon of SYFP2 was replaced by PCR with an *XbaI* site, and a *NotI* site was inserted behind the stop codon of SYFP2. The resulting fusion of GIPR-*XbaI*-SYFP2 was cloned in pcDNA3. Subsequent mutations of the linker region between receptor and SYFP2 were performed by site-directed mutagenesis (Q5 site-directed mutagenesis kit, New England Biolabs, USA). GIPR and GLP-1R both possess a putative N-terminal signal peptide that is cleaved during receptor processing and trafficking (Huang et al., 2010; Whitaker et al., 2012). Hence in order to N-terminally label the receptors, a FLAG-tag was introduced immediately downstream of the predicted signal peptide. This was achieved by replacing the myc-tag of N-terminally labeled GIPR and GLP-1R with a FLAG-tag (DYKDDDDK) by site-directed mutagenesis. Myc-tagged GIPR and GLP-1R constructs (previously described (Al-Sabah et al., 2014)) encode the influenza hemagglutinin signal peptide (MKTIIALSYIFCLVFAA) in place of the native signal peptide.

Nluc-Arr3 has been previously described (Al-Zamel et al., 2019). The whole construct was subsequently cloned into pcDNA5-FRT (Invitrogen) in order to generate a stable isogenic cell line.

mCherry-CAAX was cloned by PCR attaching the codons for the last 18 amino acids of the small GTPase H-ras to mCherry (which encode the H-ras palmitoylation site) in the process. The PCR product was cloned into pcDNA3 using *HindIII* and *NotI*. All constructs were verified through sequencing.

### Ligands

Human GLP-1 (7-36) NH2 and human GIP (1-42) were purchased from Bachem (Bubendorf, Switzerland).

### Cell Culture and Transfection

HEK-293 and Flp-In HEK-293 cells (Invitrogen) were cultured in Dulbecco’s modified Eagle’s media supplemented with 10% fetal calf serum, 100 U/ml penicillin and 100 µg/ml streptomycin. Cells were maintained at 37°C in a humidified environment containing 5% CO_2_. HEK-293 cells were transiently transfected using Effectene (Qiagen, Hilden, Germany), following the manufacturer’s protocol. In order to generate stable cell lines Flp-In HEK-293 cells were transfected with the pcDNA5.FRT vector and pOG44 using Effectene. Stable isogenic clones were selected by the addition of hygromycin at a concentration of 100 µg/ml.

### Luciferase Assay

Activation of various GIPR constructs was assessed by a luciferase reporter gene assay using a previously described protocol (Al-Sabah et al., 2014). Briefly, Flp-In HEK-293 cells were transiently transfected with cDNA encoding either GLP-1 or GIP receptor constructs and a reporter gene construct consisting of a cAMP-response element fused to a reporter gene encoding firefly luciferase (Cre-luc) using Effectene (Qiagen, Hilden, Germany), following the manufacturer’s protocol. Twenty-four hours after transfection, the cells were seeded into white 96-well plates (Thermo Scientific, Roskilde, Denmark) at a density of 10,000 cells/well. Twenty-four hours later, the cells were incubated for 3 h in media containing peptide ligand and then lysed. Luciferase activity was quantified using LucLite reagent (PerkinElmer Life and Analytic Sciences, Wellesley, MA, USA).

### Confocal Microscopy

Flp-In HEK-293 cells transiently expressing SYFP2-labeled receptors and mCherry-CAAX were plated on to a poly-d-lysin-coated coverslip and mounted on to an “Attofluor” holder (Molecular Probes, Leiden, The Netherlands). The cellular location of the labeled receptors was monitored by live cell confocal microscopy performed on a Zeiss LSM 800 meta system (Carl Zeiss, Oberkochen, Germany). Zeiss Zen Blue 2 software (2.1) was used for data acquisition and analysis. Images were taken with an oil-immersion 63× lens using the factory settings for mCherry and YFP.

### BRET Assays

Flp-INFlp-In HEK-293 cells stably expressing Arrestin3-Nluc were transiently transfected with SYFP2-labeled receptor as previously described. For BRET saturation assays increasing amounts of SYFP2-labeled receptor DNA were transfected (0–2 µg). Forty-eight hours posttransfection cells were detached and washed with Hank’s Balance Salt Solution (HBSS). Cells were re-suspended in HBBS and plated on to white 96-well plates (PerkinElmer) in suspension at a density of 180,000 cells/well. Cell were incubated with agonist for 10 min and BRET measurements were taken using a Victor X4 (PerkinElmer) plate reader immediately after the addition of coelenterazine *h* (final conc. 5 µM). Nluc emission was measured through a 460/40 nm filter and the resulting SYFP2 emission was read through a 535/25-nm filter. Expression levels of Nluc and SYFP2-labeled constructs were monitored by measuring luminescence and fluorescence respectively. Luminescence was measured using a Victor X4 and factory settings for luminescence. For fluorescence measurements, cells from the same transfection were plated on to black 96-well plates and after 1-h incubation in darkness, total fluorescence was measured with excitation 490/6 nm and an emission filter at 535/25 nm. For BRET saturation assays raw data was corrected by subtracting the BRET ratio determined from cells expressing Nluc only. Data was then plotted as BRET ratio vs. fluorescence/luminescence and curves were fitted using “one site-specific binding” function (GraphPad 7.0).

### FRET Measurements

HEK-293 cells were cotransfected with either GLP-1R-YFP or GIPR-YFP and Arr3-CFP. At 24 h posttransfection, the cells were plated on poly-D-lysine-coated coverslips (25-mm diameter) in six-well plates. After 24 h, FRET measurements were performed as previously described (Zindel et al., 2015) with two modifications: first, the light source was an LED excitation system (pE-2, CoolLED, Andover, UK); second, ligands were always applied in FRET buffer supplemented with 0.1% bovine serum albumin. The fluorescence signal at 535 nm is the sum of the YFP fluorescence and bleed through of CFP fluorescence into the YFP channel (approx. 40% of the fluorescence at 480 nm); therefore the “real” YFP fluorescence was calculated by subtracting the CFP bleed through from the F_535_ signal. FRET was calculated as F_YFP_/F_480_. To get information about the expression of YFP-tagged receptors, cells were excited directly with 500 nm light and the fluorescence intensity was measured (dYFP, d for “direct excitation”). The CFP signal before the start of the experiment is a good indication for the expression level of the arrestin (as there is no FRET at the beginning of the experiment), and therefore the ratio F_dYFP_/F_CFP_ can be used as an approximation of the stoichiometry between the receptor and arrestin.

### Data Analyses

Dose-response data were fitted to a sigmoidal curve and BRET saturation experiments were fitted to one-site specific binding curve using GraphPad 7.0 (GraphPad, San Diego, CA). The values are expressed as the mean ± standard error of the mean; *n* = number of independent experiments. Statistical analysis of significance was calculated with GraphPad 7.0 using a two-tailed, unpaired Student’s *t*-test or ANOVA where appropriate.

## Results and Discussion

### Arrestin3-Nluc Recruitment to eYFP Labeled GIPR and GLP-1R

**Figure 1:**
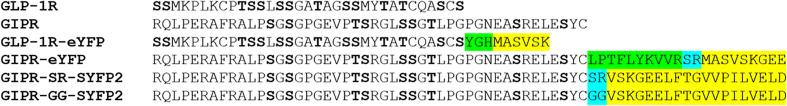
Sequence of the C-terminal region of GLP-1R and GIPR constructs used in the present study. GIPR-eYFP contains a 10–amino acid linker (shown in green) between the end of the C-terminal region and the *XbaI* site (shown in cyan), eYFP is shown in yellow. There is no linker between the end of the C-terminal tail and the *XbaI* site in GIPR-SR-SYFP2. However, this results in the introduction of a serine residue (S) at the end of GIPR’s C-terminal region which could potentially be a phosphorylation site. The SR was subsequently substituted with GG to give GIPR-GG-SYFP2.

**Figure 2:**
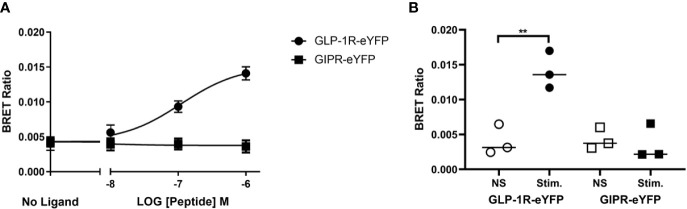
Agonist-induced bioluminescence resonance energy transfer (BRET) between receptor and arrestin3. **(A)** Dose dependent GLP-1 and GIP stimulated BRET between Nluc-tagged arrestin3 and the corresponding eYFP-labeled receptor. **(B)** Comparison of agonist (1 µM) stimulated BRET between arrestinand either GLP-1R or GIPR. The results are expressed as the mean ± standard error of the mean for at least three independent experiments; Stim., stimulated; NS, not stimulated **P < 0.01.

Using a FRET-based assay we have previously reported that activated GLP-1R tagged at the C-terminus with eYFP and expressed in HEK-293 cells interacts robustly with both CFP-tagged GRK2 and arrestin3 (β-arrestin2) whereas similarly tagged GIPR does not (Al-Sabah et al., 2014). Ismail *et al*. have also reported that GIPR does not significantly interact with either arrestin2 (β-arrestin1) or arrestin3 (Ismail et al., 2015). However, a recent study by Gabe *et al*., shows that stimulated GIPR can recruit both isoforms of arrestin (Gabe et al., 2018). Both groups used a BRET-based arrestin recruitment assay albeit using different configurations. In order to investigate this issue further we measured agonist stimulated recruitment to GIPR and GLP-1R using a BRET-based assay. Using the same receptor constructs as in our previous FRET-based experiments (GIPR-eYFP and GLP-1R-eYFP, C-terminal tail sequence shown in **Figure 1**), Flp-In HEK-293 cells stably expressing Arr3-Nluc were transfected with either GIPR-eYFP or GLP-1R-eYFP and stimulated with their corresponding peptide. GLP-1 stimulated Arr3 recruitment to GLP-1R-eYFP in a dose-dependent manner (**Figure 2A**) with a pEC_50_ value of 7.1 (± 0.3) whereas there was no significant difference in BRET ratio between unstimulated cells expressing GIPR-eYFP and those stimulated with 1 µM GIP (**Figure 2B**), confirming our previous results from FRET experiments.

### FLAG-GIPR-SR-SYFP2 Can Recruit Arr3

**Figure 3:**
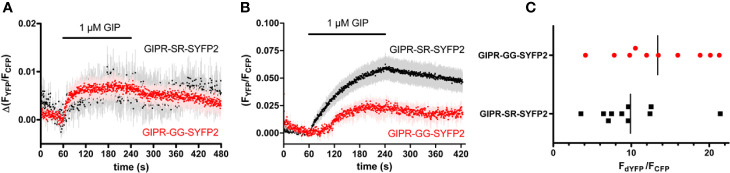
Agonist-induced fluorescence resonance energy transfer (FRET) between FLAG-GIPR-SYFP2 and GRK2 and arrestin3. **(A)** 1 µM GIP stimulated GRK2 recruitment to both FLAG-GIPR-SR-SYFP2 and FLAG-GIPR-SR-SYFP2 was detectable by FRET microscopy. The traces are the mean ± standard error of seven (SR) or eight (GG) independent experiments. **(B)** 1 µM GIP stimulated arrestin3 recruitment to FLAG-GIPR-SR-SYFP2 to a significantly (**P < 0.01) greater extent 180 s after addition of agonist than to FLAG-GIPR-GG-SYFP2. The traces are the mean ± standard error of the mean of 10 independent experiments. **(C)** Receptor (dYFP) and arrestin (CFP) expression of the experiments shown in **(B)** were quantified by measuring the CFP intensity after excitation at 430 nm and the YFP intensity after excitation at 500 nm (dYFP). The ratio of dYFP/CFP is a measure for the relative expression of the two constructs and is not significantly different between FLAGGIPR-SR-SYFP2 and Flag-GIPR-GG-SFYP.

The GIPR-eYFP construct used in the previous RET experiments contains a 10 amino acid linker between the end of the receptor’s C-terminal tail and the *XbaI* restriction-site immediately upstream of the eYFP (**Figure 1**). In subsequent FRET experiments a GIPR construct was employed that used a brighter version of YFP (SYFP2) and did not include the 10 amino acid linker found in the original construct (GIPR-SR-SYFP2 **Figure 1**). This receptor was also tagged at its N-terminus with a FLAG epitope and the native signal peptide was replaced with the influenza hemagglutinin signal peptide (FLAG-SR-GIPR-SYFP2). HEK-293 cells transiently expressing FLAG-GIPR-SR-SYFP2 and either GRK2-CFP or Arr3-CFP were observed by FRET microscopy. Stimulation with 1 µM GIP resulted in an increase in FRET ratio indicating that this GIPR construct could recruit both GRK2 and arrestin3 (**Figures 3A, B**). There are two requirements for arrestin recruitment to GPCRs; an agonist-induced change in receptor conformation and phosphorylation of the agonist-occupied receptor by G protein-coupled receptor kinases (GRKs) (Krasel et al., 2005). It has also been shown that the affinity of arrestins for a GPCR can be increased by modifying the receptor so that the number of phosphorylation sites in the C-terminal tail is increased (Zindel et al., 2015; Zhou et al., 2017). By removing the 10 amino acid linker and moving the *XbaI* restriction site, which codes for serine/arginine (SR), to the distal end of GIPR’s C-terminal tail an additional potential phosphorylation site may have been introduced to GIPR. When comparing the sequence of GIPR and GLP-1R’s C-terminal tail it can be seen that not only is GIPR’s C-terminal tail longer but that it also contains fewer serine and threonine residues. Furthermore, GLP-1R ends on a serine residue (**Figure 1**). To test if this recruitment of arrestin to GIPR was due to the presence of a potential phosphorylation site, the serine/arginine coded for by the *XbaI* site was substituted with glycine/glycine. The resulting construct, FLAG-GIPR-GG-SYFP, showed a significantly (*P*<0.01) reduced ability to recruit arrestin3 with 1 µM GIP in the FRET assay (**Figure 3B**) but nonetheless could still recruit arrestin3. Importantly there was no significant difference in the stoichiometry between YFP and CFP fluorescence for arrestin recruitment assays performed with either FLAG-GIPR-SR-SYFP or FLAG-GIPR-GG-SYFP (**Figure 3C**). Therefore, the amplitude of the FRET signal may be compared.

### GLP-1R-SYFP2, but Not GIPR-SR-SYFPR, Recruits Arrestin in a BRET Saturation Assay

**Figure 4:**
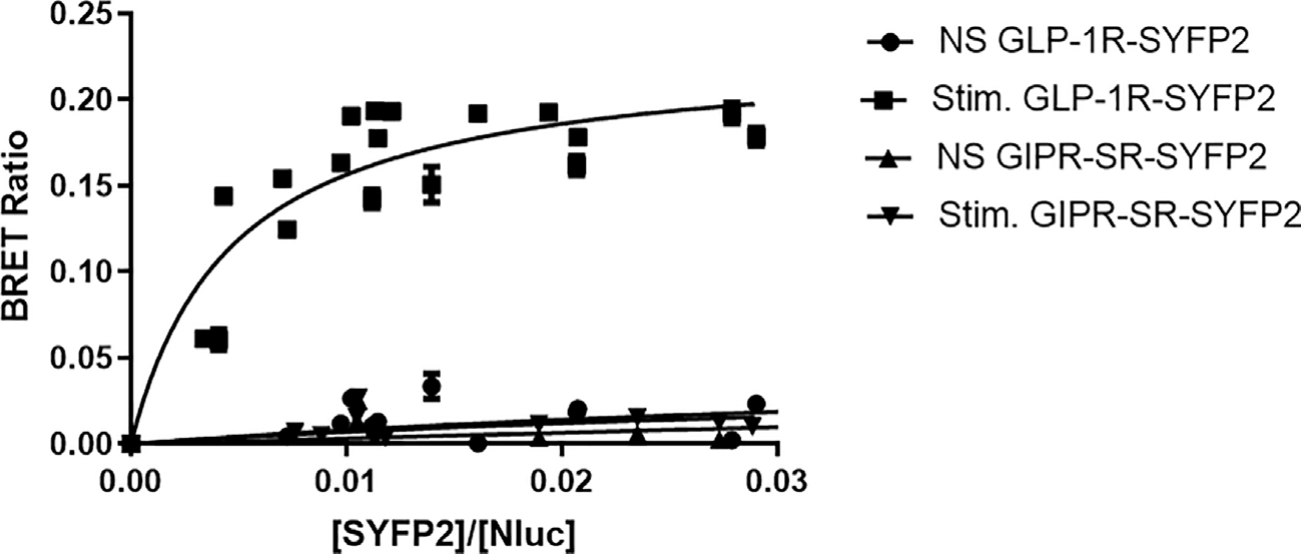
Bioluminescence resonance energy transfer (BRET) saturation experiments for agonist-induced interaction between receptor and arrestin3. Increasing amounts of SYFP2-labeled receptors were transiently expressed in Flp-In HEK-293 stably expressing Arr3-Nluc. Stimulation of GLP-1R-SYFP2 with 1 µM GLP-1 produced an exponential curve increasing and reaching an asymptote, consistent with a saturable BRET signal. All other curves increased in a quasi-linear fashion consistent with nonspecific bystander effects. Data are pooled results from at least three independent experiments performed in triplicate.

To further investigate arrestin recruitment to the incretin receptors we performed BRET saturation assays. In these experiments receptors were labeled with SYFP2 at the C-terminus (**Figure 1**) but were not tagged at the N-terminus and retain their native signal peptides. Flp-In HEK-293 cells stably expressing Arr3-Nluc were transfected with increasing amounts of either GIPR-SR-SYFP2 or GLP-1R-SYFP2 and stimulated with 1 µM of their corresponding agonist. Data were fitted to a single binding-site equation by non-linear regression. Stimulation with 1µM resulted in a BRET signal between GLP-1R-SYFP2 and Arr3-Nluc that reached saturation (BRETmax 0.22 ± 0.01) whereas the BRET signal between non-stimulated GLP-1R-SYFP2 and both stimulated and unstimulated GIPR-SR-SYFP2 and Arr-NLuc increased in a quasi-linear fashion, suggesting a non-specific interaction between receptor and arrestin (**Figure 4**). These results suggest that an additional potential phosphorylation site at the distal C-terminus is not sufficient to permit arrestin recruitment to GIPR, at least to an extent detectable in a BRET saturation assay.

### Addition of an N-Terminal FLAG-Epitope and Cotransfection of GRK2 Improve Agonist Stimulated Arrestin Recruitment to GIPR-SR-SYFP2

**Figure 5:**
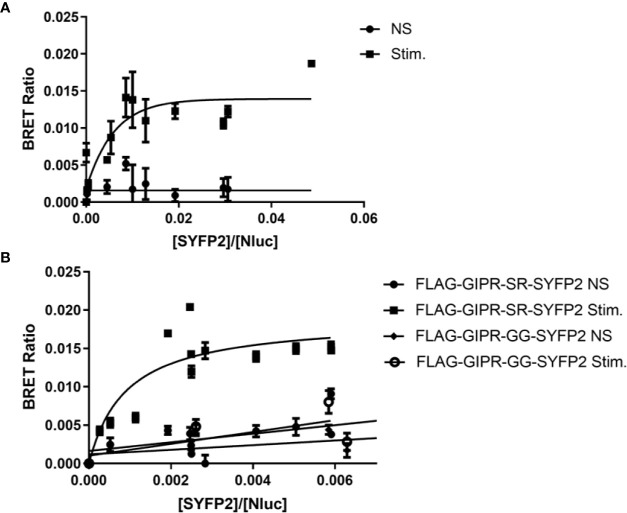
Bioluminescence resonance energy transfer (BRET) saturation experiments for agonist-induced interaction between FLAG-GIPR-SYFP2 and arrestin3. Increasing amounts of FLAG-GIPR-SYFP2 receptors were transiently expressed in Flp-In HEK-293 stably expressing Arr3-NLuc. **(A)** Stimulation of FLAG-GIPR-SR-SYFP2 with 1 µM GIP results in an increase in BRET ratio that appears to produce an exponential curve. However, this data set has a poor R square value (0.65) when fitted to a single binding-site equation. **(B)** Cotransfection of 200 ng GRK2 with the receptor results in a BRET ratio that is best described by a one-site specific-binding model when FLAG-GIPR-SR-SYFP2 is stimulated with 1 µM GIP consistent with a specific agonist-induced interaction between the receptor and arrestin3. Substitution of the SR linker with GG (FLAG-GIPR-GG-YFP2) abolishes arrestin recruitment. Data are pooled results from at least 3 independent experiments performed in triplicate.

As our FRET experiments demonstrated that FLAG-GIPR-SR-SYFP2 could recruit arrestin upon stimulation we used the same construct in a BRET saturation assay (**Figure 5A**). Although we observed an increase in BRETmax with agonist stimulation this data set had a poor R square value (0.65) when fitted to a single binding-site equation and was rejected. However, when additional GRK2 (200 ng) was cotransfected with the receptor we observed a BRET signal that was best described by a one-site specific-binding model (**Figure 5B**). Furthermore, when the potential phosphorylation site (SR) was substituted with (GG) agonist-stimulated arrestin recruitment was abolished. The results of these BRET saturation experiments are in agreement with the results of our FRET experiments for FLAG-GIPR-SR-SYFP2. However, whereas we were unable to detect arrestin recruitment to FLAG-GIPR-GG-SYFP2 in the BRET saturation assay, we were able to in the FRET assay. This discrepancy may be due to the FRET assay’s greater sensitivity or possibly because of the kinetic nature of the FRET assay. The BRET assay is an end point assay measured after a 10 min incubation with agonist whereas the FRET assay shows a peak in amplitude approximately 3 min after stimulation.

Together these data demonstrate that GIPR can be engineered to interact with arrestin under certain assay conditions. To achieve this, an additional serine residue is required at the very end of GIPR’s C-terminal tail. This was inadvertently achieved by placing an *XbaI* restriction site directly between the receptor and the fluorescent protein. However, the presence of an engineered C-terminal serine residue was not sufficient to observe arrestin recruitment to GIPR as demonstrated in our initial BRET saturation experiments (**Figure 4**). It was only with the inclusion of an N-terminal FLAG epitope and replacement of the signal sequence with that of hemagglutinin that we began to observe arrestin recruitment (**Figure 5A**) and even then the BRET signal could only reliably be fitted to a one-site specific binding curve with the cotransfection of additional GRK2 (**Figure 5B**). There are five non-visual members of the GRK family that are now understood to regulate cell signaling independent of GPCRs as well as phosphorylating GPCRs (Gurevich and Gurevich, 2019). The present study could be extended by investigating the effect of overexpression of different members of the GRK family on arrestin recruitment to both GLP-1R and GIPR. It would be interesting to investigate how the rate of arrestin association to the receptor is influenced by different GRKs.

### Substitution of GIPR’s Native Signal Peptide With the Influenza Hemagglutinin Signal Peptide Increase Receptor Expression at the Plasma Membrane

**Figure 6:**
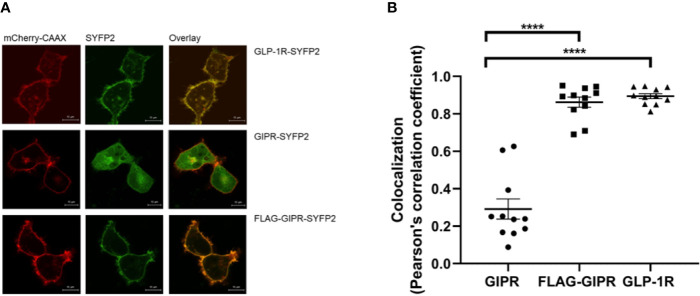
Visualization of the cellular location of SYFP2-labeled receptors transiently expressed in HEK-293 cells by confocal microscopy. **(A)** Representative live cell images of HEK-293 cells transiently cotransfected with plasma membrane targeted mCherry-CAAX (red) and SYFP2-labeled receptor (yellow). GLP-1R-SYFP2 appears to be expressed predominantly at the plasma membrane whereas GIPR-SYFP2 appears to be located not only at the plasma membrane but also in intracellular compartments. The addition of a N-terminal FLAG-epitope and substitution of the native signal peptide with the influenza hemagglutinin signal peptide appears to enhance the trafficking of GIPR to the plasma membrane. The images are representative of at least 3 independent experiments. Scale bar = 10 µm. **(B)** Colocalization of the SYFP2-labeled receptors with plasma membrane-targeted mCherry. Replacement of GIPR’s native signal peptide with the influenza hemagglutinin signal peptide significantly (****P < 0.001) increases the receptor’s colocalization with membrane-targeted mCherry. GLP-1R-SYFP2 with its native signal peptide colocalizes with membrane-targeted mCherry to a significantly (****P < 0.001) greater extent than GIPR-SYFP2 with its native signal peptide. Data are the mean ± SEM from values measured in *n* = 11.

The influenza hemagglutinin signal peptide has been shown to enhance the surface expression of the β_2_-adrenergic receptor and is often used for this purpose when studying GPCRs in a recombinant system (Guan et al., 1992). When expressed in adipocytes GIPR has been shown to be constitutively trafficked between the plasma membrane and intracellular compartments with less than half of the receptors being expressed at the cell membrane (Mohammad et al., 2014). We investigated the effect of replacing the native signal peptide with that of the hemagglutinin signal peptide on GIPR’s cell surface expression further. HEK-293 cells transiently expressing the YFP2-labeled receptor and a membrane-targeted red fluorescent protein (mCherry) were observed by confocal microscopy. GLP-1R-SYFP2 appeared to be expressed predominantly at the plasma membrane whereas GIPR-SYFP2 appeared to also be located in intracellular compartments. The addition of a N-terminal FLAG-epitope and substitution of the native signal peptide with the influenza hemagglutinin signal peptide appears to enhance the trafficking of GIPR to the plasma membrane (**Figure 6A**) and significantly increased (*P*<0.001) the receptor’s colocalization with membrane-targeted mCherry (**Figure 6B**). This observation may be more accurately quantified by ligand binding studies. Nonetheless, the data from the confocal microscopy experiments demonstrate that the proportion of expressed GIPR that colocalizes with plasma membrane-targeted mCherry-CAAX is increased when the native signal peptide is replaced with the hemagglutinin signal peptide.

To test if the addition of the YFP variants to the C-terminus affects the ability of GIPR to signal through Gs, dose response curves were generated using a cAMP-response element-linked reporter gene assay (**Table 1**). GIP displayed a similar potency at all constructs used in the present study except GIPR-SYFP2 (SR) where GIP was significantly less potent than at wild type (*P*<0.01) and FLAG-GIPR-SYFP2 (GG) (*P*<0.05). This could possibly be explained by the absence of the hemagglutinin signal peptide and reduced receptor surface expression. Nonetheless, all receptor constructs retained their ability to interact with Gs including those that showed no interaction with arrestin3 (GIPR-eYFP, FLAG-GIPR-GG-SYFP2 and GIPR-SR-SYFP2).

**Table 1:** Activation of GIPR constructs used in this study by Glucose-dependent insulinotropic polypeptide (GIP).

Receptor	*p*EC_50_
Wild Type GIPR	10.8 ± 0.35_(3)_
GIPR-eYFP	9.7 ± 0.19_(3)_
FLAG-GIPR-SR-SYFP2	9.9 ± 0.03_(3)_
FLAG-GIPR-GG-SYFP2	10.1 ± 0.51_(3)_
GIPR-SR-SYFP2	8.6 ± 0.19_(3)_a, b

The mean ± SEM shown are from at least three independent experiments (the number of experiments is shown in parentheses). pEC_50_ refers to the –log EC_50/_M. (a) P<0.01, (b) P<0.05 significantly different from wild type GIPR and FLAG-GIPR-GG-SYFP2 (GG) respectively. Statistical analysis tests were performed using Graph Pad 7.0 and analysis of variance was followed by a post hoc test (Turkey).

## Conclusion

GPCRs such as the β3-adrenergic receptor (β3AR) and the gonadotropin-releasing hormone (GnRHR) are known not to interact with arrestin (Cao et al., 2000; Jensen et al., 2013). While GLP-1R has been shown to robustly recruit arrestin, the literature regarding GIPR’s ability to bind arrestin is contradictory (Al-Sabah et al., 2014; Ismail et al., 2015; Gabe et al., 2018). The results presented here demonstrate how arrestin recruitment assay configuration can dramatically alter the result. Substitution of the native signal peptide with the influenza hemagglutinin signal peptide influences the outcome of the experiment, most likely by enhancing cell surface expression of the receptor. Our results also highlight the importance of the linker between receptor and fluorescent protein in BRET and FRET-based arrestin recruitment assays. The use of an *XbaI* restriction site may introduce an additional potential phosphorylation site, resulting in false positive results. GPCRs are also commonly tagged at their C-terminal tails with a 1D4 epitope (TETSQVAPA) (Cai et al., 2017). This epitope also adds potential phosphorylation sites to the receptors’ C-terminal tail and may also affect the results of arrestin recruitment assays. With the advent of the concept of “biased agonism” and “functional selectivity” these observations become pertinent to drug discovery programs.

## Data Availability Statement

The raw data supporting the conclusions of this article will be made available by the authors, without undue reservation.

## Author Contributions

Conceptualization: SA-S and CK. Formal analysis: SA-S and CK. Investigation: SA-S, LA, MB, and CK. Writing—original draft: SA-S. Writing—review and editing: SA-S, MB, and CK.

## Conflict of Interest

The authors declare that the research was conducted in the absence of any commercial or financial relationships that could be construed as a potential conflict of interest.
